# Progressive structural and covariance connectivity abnormalities in patients with Alzheimer’s disease

**DOI:** 10.3389/fnagi.2022.1064667

**Published:** 2023-01-06

**Authors:** Yaqiong Xiao, Jiaojian Wang, Kaiyu Huang, Lei Gao, Shun Yao, for the Alzheimer’s Disease Neuroimaging Initiative

**Affiliations:** ^1^Center for Language and Brain, Shenzhen Institute of Neuroscience, Shenzhen, China; ^2^State Key Laboratory of Primate Biomedical Research, Institute of Primate Translational Medicine, Kunming University of Science and Technology, Kunming, China; ^3^Department of Radiology, Zhongnan Hospital of Wuhan University, Wuhan, China; ^4^Department of Neurosurgery, The First Affiliated Hospital, Sun Yat-sen University, Guangzhou, China

**Keywords:** Alzheimer’s disease, grey matter atrophy, progressive changes, superior temporal regions, left caudate, cognitive decline

## Abstract

**Background:**

Alzheimer’s disease (AD) is one of most prevalent neurodegenerative diseases worldwide and characterized by cognitive decline and brain structure atrophy. While studies have reported substantial grey matter atrophy related to progression of AD, it remains unclear about brain regions with progressive grey matter atrophy, covariance connectivity, and the associations with cognitive decline in AD patients.

**Objective:**

This study aims to investigate the grey matter atrophy, structural covariance connectivity abnormalities, and the correlations between grey matter atrophy and cognitive decline during AD progression.

**Materials:**

We analyzed neuroimaging data of healthy controls (HC, *n* = 45) and AD patients (*n* = 40) at baseline (AD-T1) and one-year follow-up (AD-T2) obtained from the Alzheimer’s Disease Neuroimaging Initiative. We investigated AD-related progressive changes of grey matter volume, covariance connectivity, and the clinical relevance to further understand the pathological progression of AD.

**Results:**

The results showed clear patterns of grey matter atrophy in inferior frontal gyrus, prefrontal cortex, lateral temporal gyrus, posterior cingulate cortex, insula, hippocampus, caudate, and thalamus in AD patients. There was significant atrophy in bilateral superior temporal gyrus (STG) and left caudate in AD patients over a one-year period, and the grey matter volume decrease in right STG and left caudate was correlated with cognitive decline. Additionally, we found reduced structural covariance connectivity between right STG and left caudate in AD patients. Using AD-related grey matter atrophy as features, there was high discrimination accuracy of AD patients from HC, and AD patients at different time points.

## Introduction

Alzheimer’s disease (AD) is one of most prevalent neurodegenerative diseases and characterized by brain atrophy in a variety of regions as revealed by non-invasive magnetic resonance imaging (MRI) technique. These regions included bilateral hippocampus, temporal lobes, superior and lateral temporal gyrus, parietal lobe, anterior and posterior cingulate cortices, thalamus, entorhinal cortex, and cerebellum (Baron et al., 2001; Busatto et al., 2003; Guo et al., 2010; Chapleau et al., 2016; Dicks et al., 2019; Kang et al., 2019; Van De Mortel et al., 2021). While early studies mainly focused on one clinical group of AD patients (Baron et al., 2001; Busatto et al., 2003; Guo et al., 2010), a growing number of recent explorations investigated brain atrophy in patients at different stages of cognitive decline (Thomann et al., 2008; Toniolo et al., 2018; Dicks et al., 2019; Van De Mortel et al., 2021). For example, a study including early and late mild cognitive impairment (EMCI, LMCI) and AD patients reported widespread and progressive grey matter atrophy, especially in bilateral temporal areas, hippocampus, and thalamus, in AD patients as compared to cognitively normal individuals (Van De Mortel et al., 2021). Moreover, a few longitudinal studies have shown progressive grey matter atrophy in AD patients over a one-year period (Anderson et al., 2012; Dicks et al., 2019). However, still little is known regarding the brain regions with progressive grey matter atrophy and the associations between the grey matter atrophy and rapid cognitive decline in AD patients.

Notably, a growing number of studies have also characterized the synchronous brain atrophy in ASD patients by employing a structural covariance network (SCN) approach (Hafkemeijer et al., 2016; Montembeault et al., 2016; Chang et al., 2018, 2021; Li et al., 2019). The SCN approach is essentially a correlation analysis for cross-sectional morphometric imaging data, which measures the synchronized grey matter atrophy undergoing common pathological processes between brain regions (Seeley et al., 2009; Alexander-Bloch et al., 2013; Evans, 2013). By investigating the underlying structural abnormalities from a network perspective, the SCN approach may provide additional valuable information to the pathology beyond the traditional voxel-based approach that focuses on local grey matter atrophy (Evans, 2013). It has been shown that there was reduced structural covariance connectivity between regions within the default mode network (DMN) in AD patients as compared to control adults (Spreng and Turner, 2013; Montembeault et al., 2016). Research has also investigated the SCN changes in different neuropathological stages of AD. It reported increased structural covariance connectivity at the early stage and decreased structural covariance connectivity at the late stage for both the DMN and the salience network, but continuously increased structural covariance connectivity for the executive control network along the AD neuropathological progression (Li et al., 2019). So far, however, no studies have investigated the progressive changes of SCN associated with the grey matter atrophy in AD patients.

Recently, a causal SCN approach, i.e., causal relationships between morphometric features of different brain regions, has been proposed to better understand the underlying structural abnormalities related to AD progression, as it is likely that the atrophy in some brain regions has a causal influence on the atrophy of other regions (Zhang et al., 2017). In a recent study, causal SCN was evaluated in MCI and AD patients using the Granger causality analysis, and this study reported the atrophy in hippocampus, thalamus, and precuneus/posterior cingulate cortex had causal effects on the atrophy of other regions during AD progression (Qing et al., 2021). These findings demonstrate causal relationships among the brain atrophy in different regions related to the progression of AD. However, the causal SCN has not been examined longitudinally in AD patients. Thus, it is still unknown whether and how earlier brain atrophy patterns impact the progressive changes of grey matter atrophy in AD patients over time.

To fill in these gaps, in the present study, we first investigated the alternations in grey matter volume (GMV) in a cohort of AD patients at different time points as compared to healthy controls (HC). Here, to better detect AD-related GMV changes, we used a relatively new approach, i.e., grey matter based spatial statistics (Ball et al., 2013; Parvathaneni et al., 2017; Ouyang et al., 2019), which measures the GMV at the core of the cortical plate and thus alleviates partial volume effect and minimizes individual variability (Ouyang et al., 2019). Then, we examined the overlapped decrease and continuing decrease of GMV in AD patients as compared to HC, and progressive decrease of GMV in AD patients between different time points. We expected reduced GMV in AD patients at both time points as compared to HC and progressive decrease in GMV during AD progression. We tested whether the grey matter atrophy in AD patients would be associated with cognitive decline over a one-year period. To further understand the brain morphological abnormalities related to the AD pathological progression, we examined the SCN and also causal SCN between the regions showing grey matter atrophy, and hypothesized that there would be reduced SCN in AD patients. Finally, we explored whether these AD-related grey matter changes could serve as biomarkers to distinguish AD patients at different stages and HC using a linear support vector machine (SVM) approach.

## Materials and methods

### Participants

The data included in this study were obtained from the Alzheimer’s Disease Neuroimaging Initiative (ADNI) database.^1^ The ADNI was launched in 2003 as a public-private partnership, led by Principal Investigator Michael W. Weiner, MD. The primary goal of ADNI has been to test whether serial MRI, positron emission tomography (PET), other biological markers, and clinical and neuropsychological assessment can be combined to measure the progression of MCI and early AD. For more details, see www.adni-info.org and previous publications (Weiner et al., 2010, 2017; Aisen et al., 2015).

Specifically, we included 40 AD subjects (22 males; mean age at baseline: 74.66 ± 7.23 years, range 56–88 years; mean age at one-year follow-up: 75.73 ± 7.25 years, range 57.0–89.3 years) and 45 HC (27 males; mean age 74.78 ± 4.95 years, range 66.6–84.8 years) in the present study. All the participants had demographic information (i.e., age, gender), clinical, and cognitive measures including Clinical Dementia Rating (CDR) (Morris, 1993) and Mini Mental State Examination (MMSE) (Folstein et al., 1975). All the AD patients at baseline (AD-T1, *n* = 40) and the majority of AD patients at one-year follow-up (AD-T2, *n* = 38) and HC (*n* = 43) completed the Montreal Cognitive Assessment (MoCA; Nasreddine et al., 2005). For the detailed demographics and clinical scores of all subjects, see Table 1.

**Table 1:** Demographic characteristics and clinical scores of AD patients at different time points (AD-T1, AD-T2) and healthy controls (HC).

	AD-T1 (*n* = 40)		AD-T2 (*n* = 40)		HC (*n* = 45)		*p* value	
	Mean ± SD	Range	Mean ± SD	Range	Mean ± SD	Range	HC vs. AD-T1	HC vs. AD-T2
Gender (M/F)	22/18		22/18		27/18		0.81^a^	0.81^a^
Age (years)	74.66 ± 7.23	56–88	75.73 ± 7.25	57–89.3	74.78 ± 4.95	66.6–84.8	0.92	0.49
CDR	0.76 ± 0.32	0.5–2.0	0.94 ± 0.47	0.5–2.0	0 ± 0	0–0	<0.001	<0.001
MMSE	23.1 ± 2.06	19–26	21 ± 4.5	9–29	29.13 ± 1.25	24–30	<0.001	<0.001
MoCA^*^	18.65 ± 4.62	11–27	16.58 ± 5.65	7–27	24.35 ± 2.46	18–29	<0.001	<0.001

Abbreviations: AD, Alzheimer‘s disease; HC, Healthy control; CDR, Clinical Dementia Rating; MMSE, Mini-Mental State Examination; MoCA, Montreal Cognitive Assessment.

a *p* value of chi-squared test.

* *n* = 38 AD patients (AD-T2) and *n* = 43 HC participants completed the MoCA.

### MRI data collection

High-resolution 3D structural images were acquired from 3 T SIEMENS scanners with a T1-weighted, magnetization prepared rapid gradient-echo (MPRAGE) sequence (TR/TI = 2300/900 ms, TE = 2.98 ms, FOV = 240 mm, 1 × 1 × 1.2 mm^3^, flip angle = 9°, Slice Thickness = 1.2 mm). Raw Digital Imaging and Communications in Medicine (DICOM) MRI scans were downloaded from the public ADNI site,^2^ reviewed for quality, and automatically corrected for spatial distortion caused by gradient nonlinearity and B1 field inhomogeneity.

### MRI data preprocessing

Voxel-based morphometry (VBM) was performed using a conventional method with Statistical Parametric Mapping (SPM8, http://www.fil.ion.ucl.ac.uk), running on Matlab 2014b (MathWorks, Natick, MA, United States). First, MR images were visually inspected and then segmented into grey matter, white matter, and cerebrospinal fluid using SPM8’s standard unified segmentation module with the default tissue probability maps (Ashburner and Friston, 2005). Second, a study-specific grey matter template was derived from the entire image dataset using the diffeomorphic anatomical registration using lie algebra (DARTEL) (Ashburner, 2007). Third, after an initial affine registration of the DARTEL template to the grey matter tissue probability map, nonlinear warping of the segmented images was then performed to match the MNI space. Finally, for the intensity preservation of the grey matter concentration, normalized grey matter images were modulated with Jacobian determinants from the normalization procedure.

### Measurement of GMV on the cortical skeleton

The GMV was measured on the cortical skeleton, that is, the center of the cortical plate, which is supposed to alleviate partial volume effects and enhance the measurement accuracy (Ouyang et al., 2019). To obtain the gray matter skeleton, the mean grey matter image was calculated across all the subjects including AD patients (both AD-T1 and AD-T2) and HC. Next, the cortical skeleton was created by applying the skeletonization function (i.e., “tbss_skeleton”) in TBSS of FSL^3^ to the averaged grey matter map. In this way, GMV was measured at the core cortical regions.

### Group differences in GMV on the cortical skeleton

First, we examined the global changes in total GMV on the cortical skeleton between HC, AD-T1, and AD-T2 groups. The total GMV was extracted for each group separately, and then two-tailed two-sample *t*-tests (HC vs. AD-T1, HC vs. AD-T2) and two-tailed paired *t*-test (AD-T1 vs. AD-T2) were conducted to test the group differences.

Then, we examined the group differences in grey matter skeleton at the voxel-level between AD-T1 and HC and between AD-T2 and HC by conducting two-tailed two-sample *t*-tests. We further identified the overlapped decrease areas that showed significant differences in the comparison of both AD-T1 vs. HC and AD-T2 vs. HC, and continuing decrease areas that showed significant differences between the comparison of AD-T1 vs. HC and AD-T2 vs. HC. The resulting maps were corrected for multiple comparisons using the FDR method with *p* < 0.05.

### Progressive changes in AD patients and brain-behavior correlation analysis

We tested the progressive GMV changes in AD patients between two time points (i.e., AD-T2 vs. AD-T1) using two-tailed paired *t*-test. The significant clusters were corrected for multiple comparisons using the FDR method with *p* < 0.05.

Subsequently, we examined the clinical relevance of significant GMV changes between AD-T2 and AD-T1. Specifically, we extracted the mean GMV values within each region showing significant changes and calculated the change GMV values (AD-T2 - AD-T1) for each region which were then correlated with changes in the cognitive ability as measured by the MoCA (i.e., change scores of the MoCA between AD-T2 and AD-T1).

### Structural covariance connectivity analysis

To examine whether and how regions showing significant grey matter atrophy were co-varied with each other, we calculated the structural covariance connectivity between these brain regions. First, overlapped decrease areas in AD patients at both time points vs. HC, continuing decrease areas from comparisons between AD-T2 vs. HC and AD-T1 vs. HC, and regions showing progressive decrease between AD-T2 and AD-T1, were selected as seed regions for covariance connectivity analysis. Next, mean GMV values within each seed region was calculated. Then, the correlations between mean GMV values of any two seed regions were computed using Pearson’s correlation coefficient. To identify abnormalities and progressive changes in the structural covariance connectivity in AD patients, we used a nonparametric permutation test to test the statistical significance of the between-group differences. We performed permutation tests with 10,000 permutations and recorded all of the differences between the two groups. Finally, we reported whether the between-group difference in the real structural covariance connectivity was out of 95% (two-tailed) of the supposed between-group differences.

In an exploratory analysis, we investigated the causal SCN using the Granger causal analysis. Specifically, we examined the causal effects of GMV within overlapped decrease areas in AD-T1 on GMV changes in AD-T2 (including continuing decrease areas and regions showing progressive changes). Since there was no clear chronological information for all the enrolled subjects, we performed the permutation test with 10,000 permutations to test the stability of the casual effects and recorded all of the causal correlations between GMV of the overlapped decrease areas in AD-T1 and GMV of continuing decrease areas and regions with progressive changes in AD-T2. The distribution and mean causal correlation values from 10,000 permutations were depicted and the significant level (set as *p* < 0.05) was determined by comparing all the permutated causal coefficients with zeros.

### Classification analysis

We applied a linear SVM approach to classify AD patients and HC using AD-based grey matter atrophy as features. Specifically, we calculated the mean GMV values within (a) overlapped decrease areas in AD patients at both time points vs. HC, (b) continuing decrease areas from comparisons between AD-T2 vs. HC and AD-T1 vs. HC, and (c) regions showing progressive decrease between AD-T2 and AD-T1. The mean GMV values of these regions were features for the classification analysis. And for the SVM analysis, we used the Matlab version of LIBSVM (Chang and Lin, 2011), a library for support vector machines, and the leave-one-out cross-validation (LOOCV) technique for SVM classifier validation. Given labeled training data, the SVM algorithm yielded an optimal hyperplane, which was applied to the classification of AD-T1 from HC, AD-T2 from HC, and AD-T1 from AD-T2, separately. The trained SVM’s classification performance was assessed using the following measures: accuracy, sensitivity, and specificity.

## Results

### Overall grey matter atrophy in AD patients

**Figure 1:**
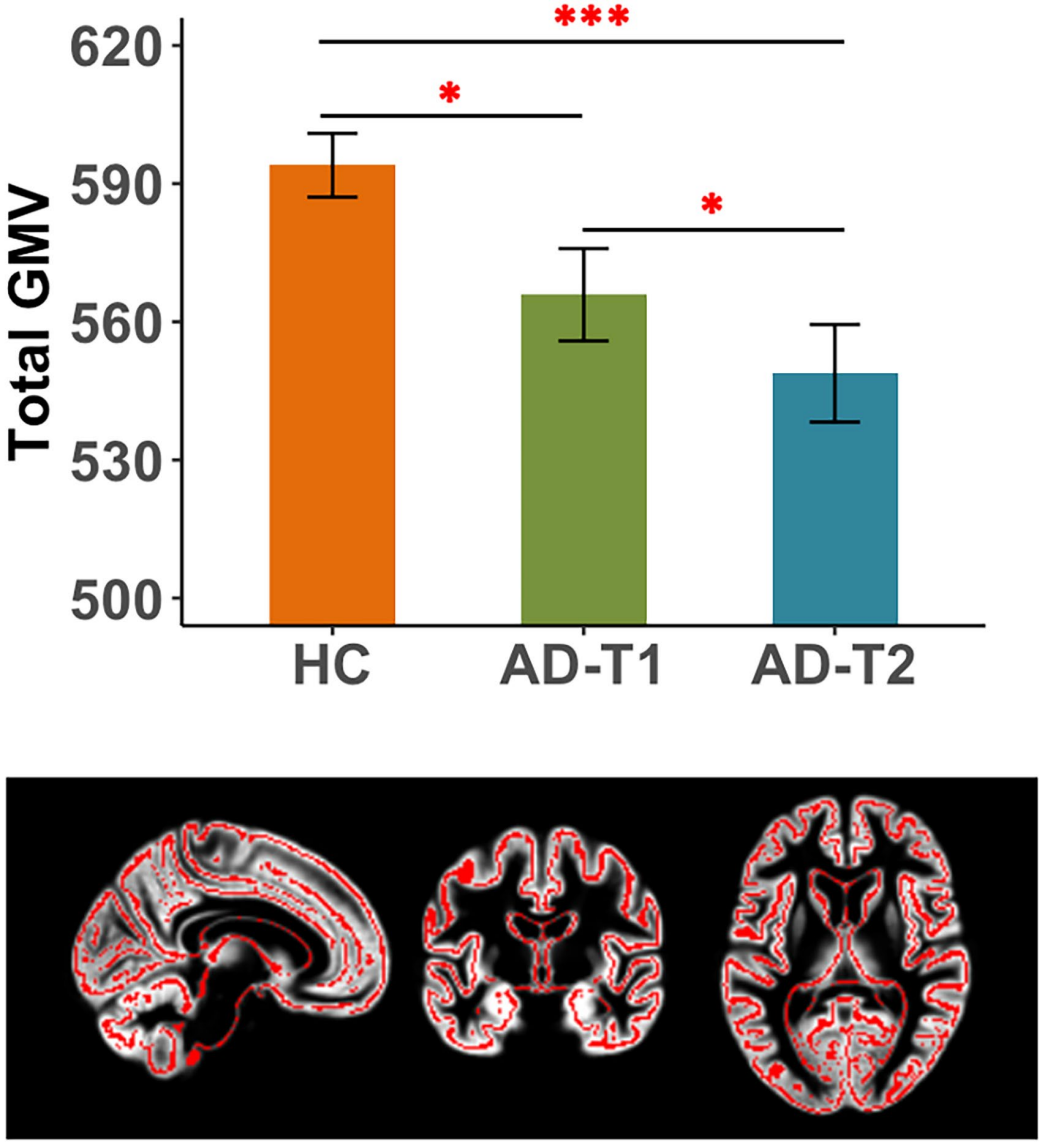
Reduced total grey matter volume (GMV) in AD patients. There were significant differences in total GMV between HC and AD-T1, between HC and AD-T2, and between AD-T1 and AD-T2. *p < 0.05, ***p < 0.001.

There were significant differences in total GMV between HC, AD-T1, and AD-T2, i.e., the greatest GMV in HC, following by AD-T1 and then AD-T2 (Figure 1). The results were corrected for multiple comparisons with the FDR method at *p* < 0.05.

### Grey matter atrophy in AD patients

**Figure 2:**
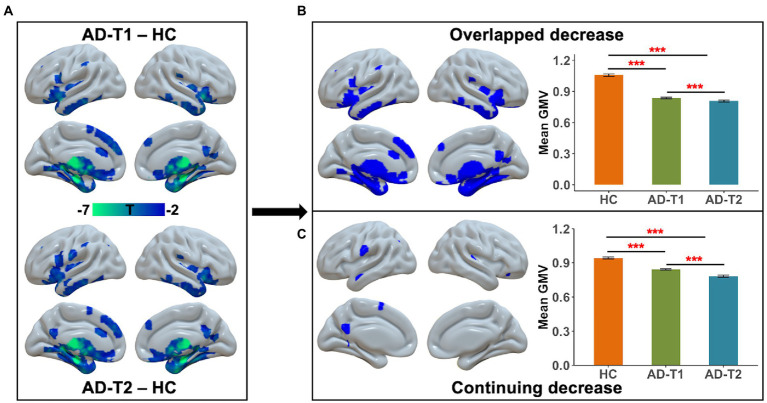
Brain areas showing substantial atrophy in grey matter volume in AD patients. **(A)** There was reduced grey matter volume in inferior frontal gyrus, prefrontal cortex, lateral temporal gyrus, posterior cingulate cortex, insula, hippocampus, caudate, and thalamus in both AD-T1 and AD-T2 as compared to HC. **(B)** The areas that showed grey matter atrophy in both AD-T1 vs. HC and AD-T2 vs. HC were identified as overlapped decrease areas, and the mean grey matter volume values within these areas are significantly different between HC and AD-T1, between HC and AD-T2, and between AD-T1 and AD-T2. **(C)** The regions that showed differences between AD-T2 vs. HC and AD-T1 vs. HC were identified as continuing decrease areas, and the mean grey matter volume values within these regions are significantly different between HC and AD-T1, between HC and AD-T2, and between AD-T1 and AD-T2. All brain maps were corrected with the FDR method (*p* < 0.05, cluster size >30 voxels). Error bars represent the standard error of the mean. ***p < 0.001.

As compared to HC, there were a wide range of regions showing significantly decreased GMV in AD patients at both time points, including inferior frontal gyrus, prefrontal cortex, lateral temporal gyrus, posterior cingulate cortex, insula, hippocampus, caudate, and thalamus (Figure 2A). These regions were identified as overlapped decrease areas, and the averaged GMV within these areas showed significant decrease between HC and AD-T1, between HC and AD-T2, and between AD-T1 and AD-T2 (Figure 2B). We further identified continuing decrease areas by comparing the differences between AD-T2 vs. HC and AD-T1 vs. HC, which included posterior cingulate cortex, ventral precentral gyrus, and lateral temporal gyrus; there was significant decrease in AD patients at both time points vs. HC and in AD-T2 vs. AD-T1 (Figure 2C).

### Grey matter atrophy in AD patients and the clinical relevance

**Figure 3:**
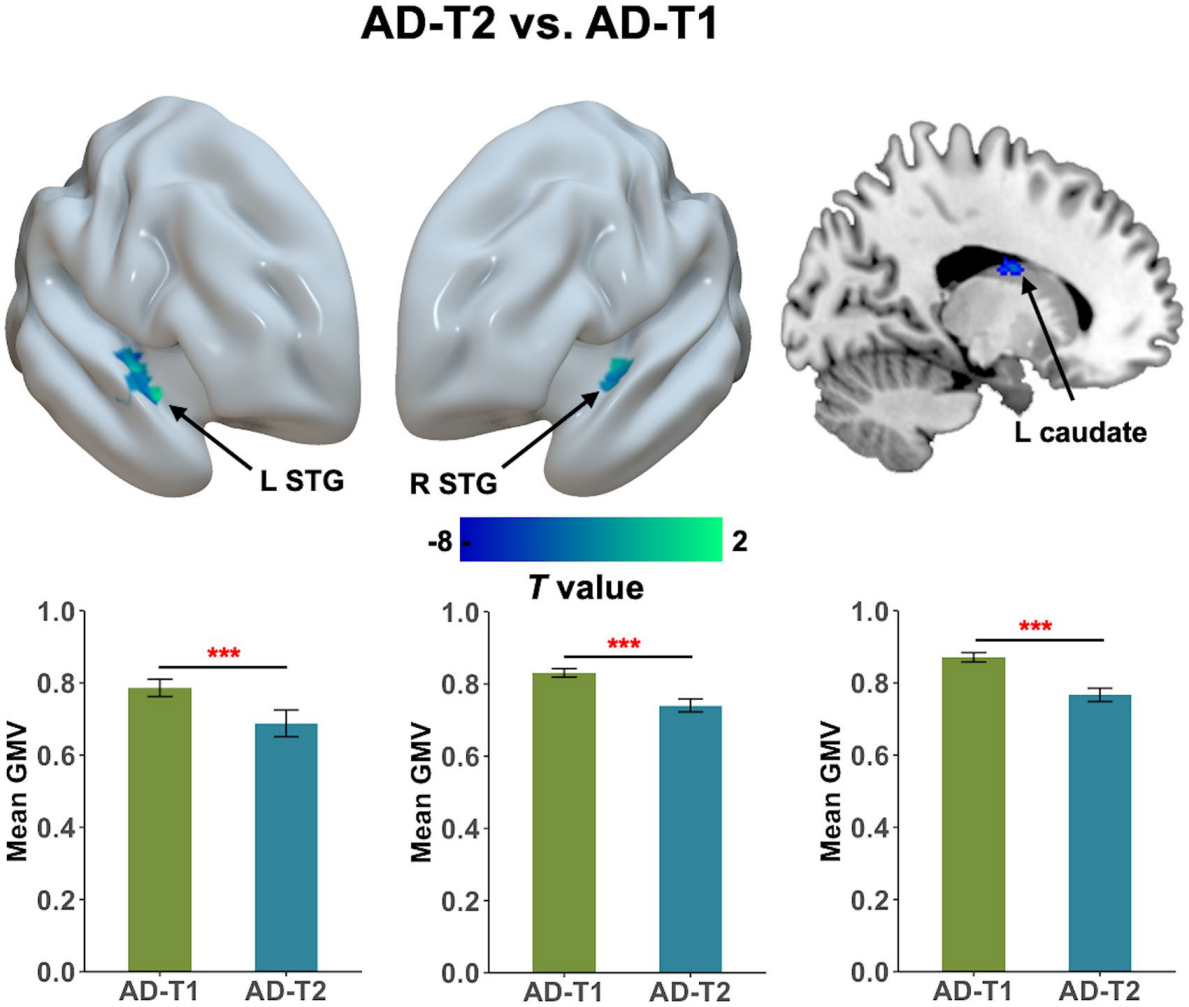
Progressive atrophy in grey matter volume between AD-T2 and AD-T1. There was significant decrease in bilateral STG and left caudate in AD patients over a one-year period. Error bars represent the standard error of the mean. Error bars represent the standard error of the mean. L, left; R, right. ***p < 0.001.

**Figure 4:**
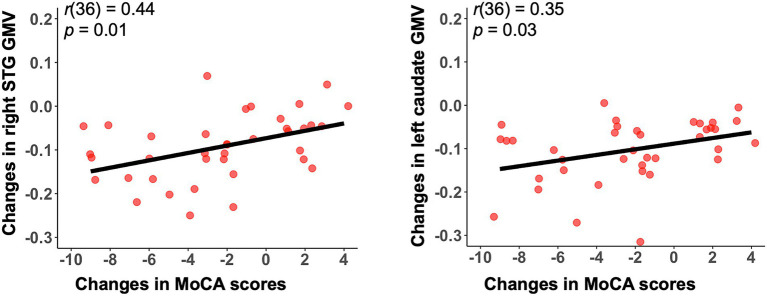
Significant correlations between change scores in the MoCA and mean GMV changes in both right STG and left caudate. Changes indicate differences between AD-T2 and AD-T1. GMV, grey matter volume; STG, superior temporal gyrus. MoCA, Montreal Cognitive Assessment.

As shown in Figure 3, we observed significant grey matter atrophy in bilateral STG and left caudate in AD-T2 as compared to AD-T1 (FDR corrected *p* < 0.05, cluster size >30 voxels). Further, we found significantly positive correlations between change scores of the MoCA and change values of GMV in both right STG [*r*(36) = 0.44, *p* = 0.01] and left caudate [*r*(36) = 0.35, *p* = 0.03; Figure 4], but not in left STG [*r*(36) = 0.25, *p* = 0.12].

### Disrupted structural covariance connectivity patterns in AD patients

**Figure 5:**
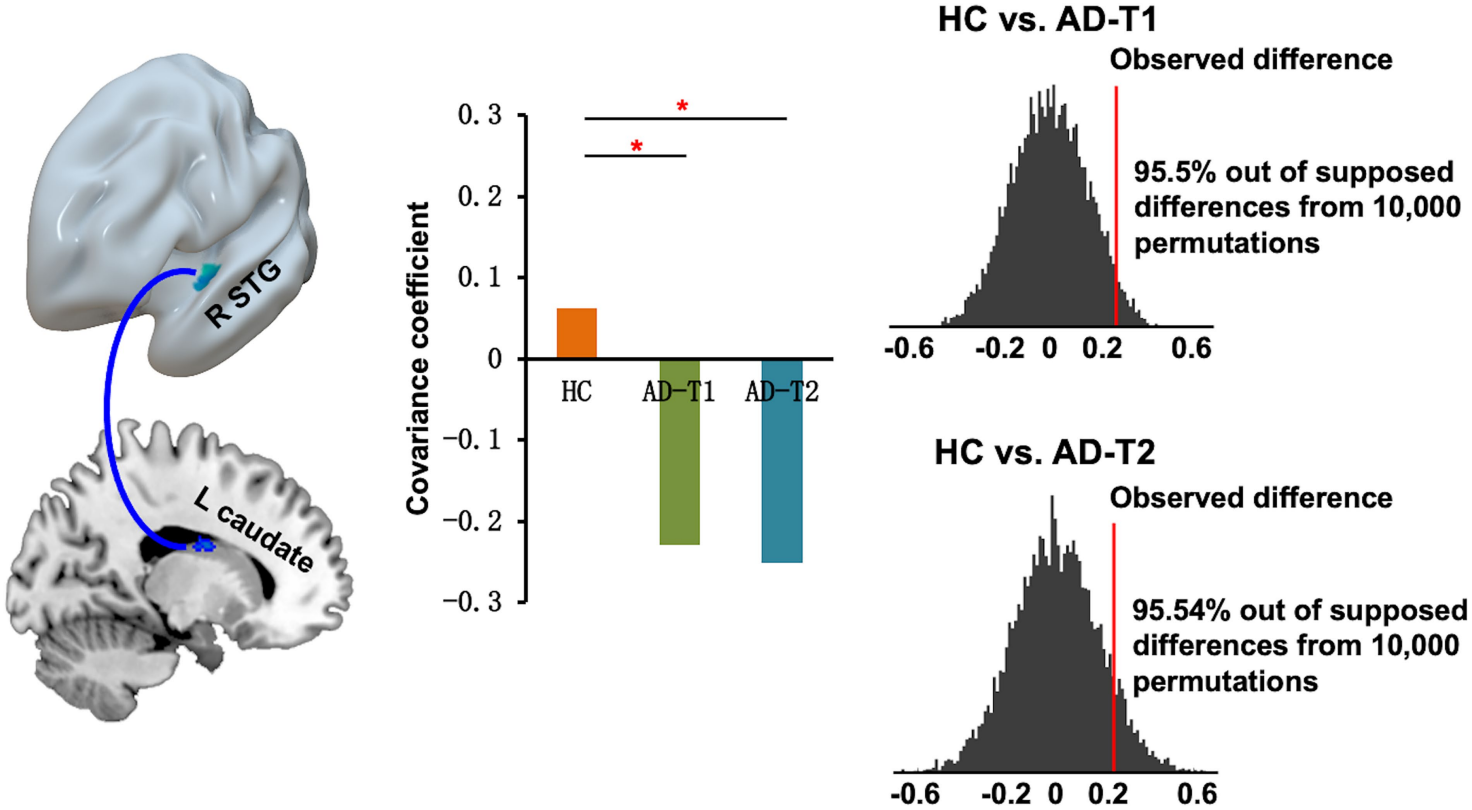
Decreased structural covariance connectivity between right STG and left caudate in AD patients at both time points as compared to HC. The permutation tests demonstrated that the between-group difference in the real covariance connectivity was out of 95% (two-tailed) of the supposed between-group differences. The red lines indicate observed difference in the strength of correlations between STG volume and left caudate volume for HC vs. AD-T1 and HC vs. AD-T2. *p < 0.05.

The structural covariance connectivity analysis showed significantly decreased strength of correlation between mean GMV values of the right STG and the left caudate in AD patients at both time points as compared to HC (Figure 5). The significant results were revealed by the permutation tests, which demonstrated the real structural covariance connectivity was out of 95% (two-tailed) of the supposed between-group differences.

**Figure 6:**
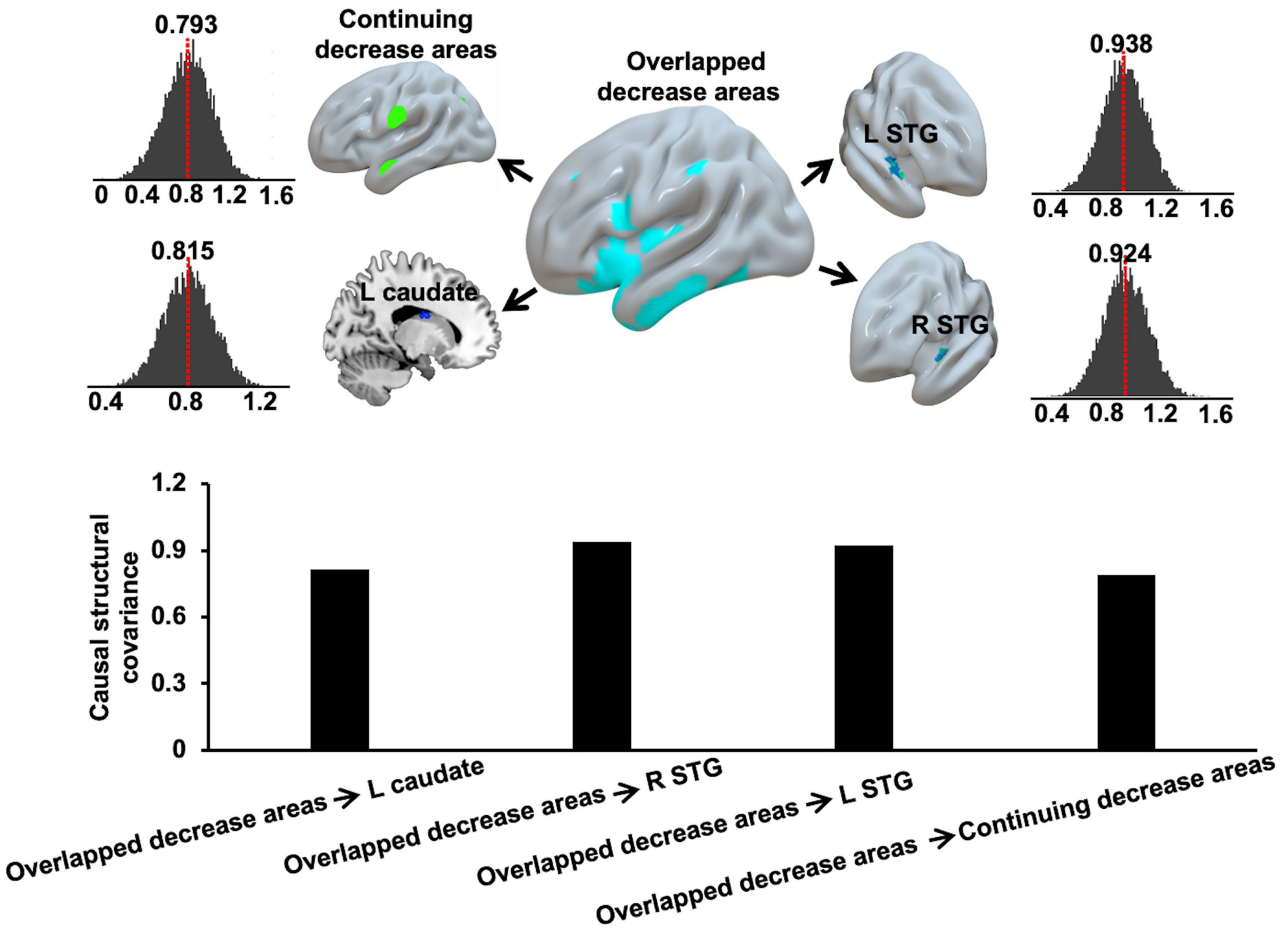
Causal effects of overlapped decrease areas in AD-T1 on the continuing decrease areas, left and right STG, and left caudate in AD-T2. The red dash lines and the bar plots indicate the mean causal correlation between GMV of the overlapped decrease areas and GMV of continuing decrease areas, left and right STG, and left caudate in AD-T2 across 10,000 permutations.

The causal SCN analysis revealed that overlapped decrease areas in AD-T1 had strong causal effects on the continuing decrease areas, bilateral STG, and left caudate in AD-T2. The causal effects were defined as the mean causal correlation between GMV of the overlapped decrease areas and GMV of continuing decrease areas, bilateral STG, and left caudate in AD-T2 across 10,000 permutations, which were significant (*p* < 0.05) as tested by comparing all the permutated causal coefficients with zero (Figure 6).

### SVM classification results

**Figure 7:**
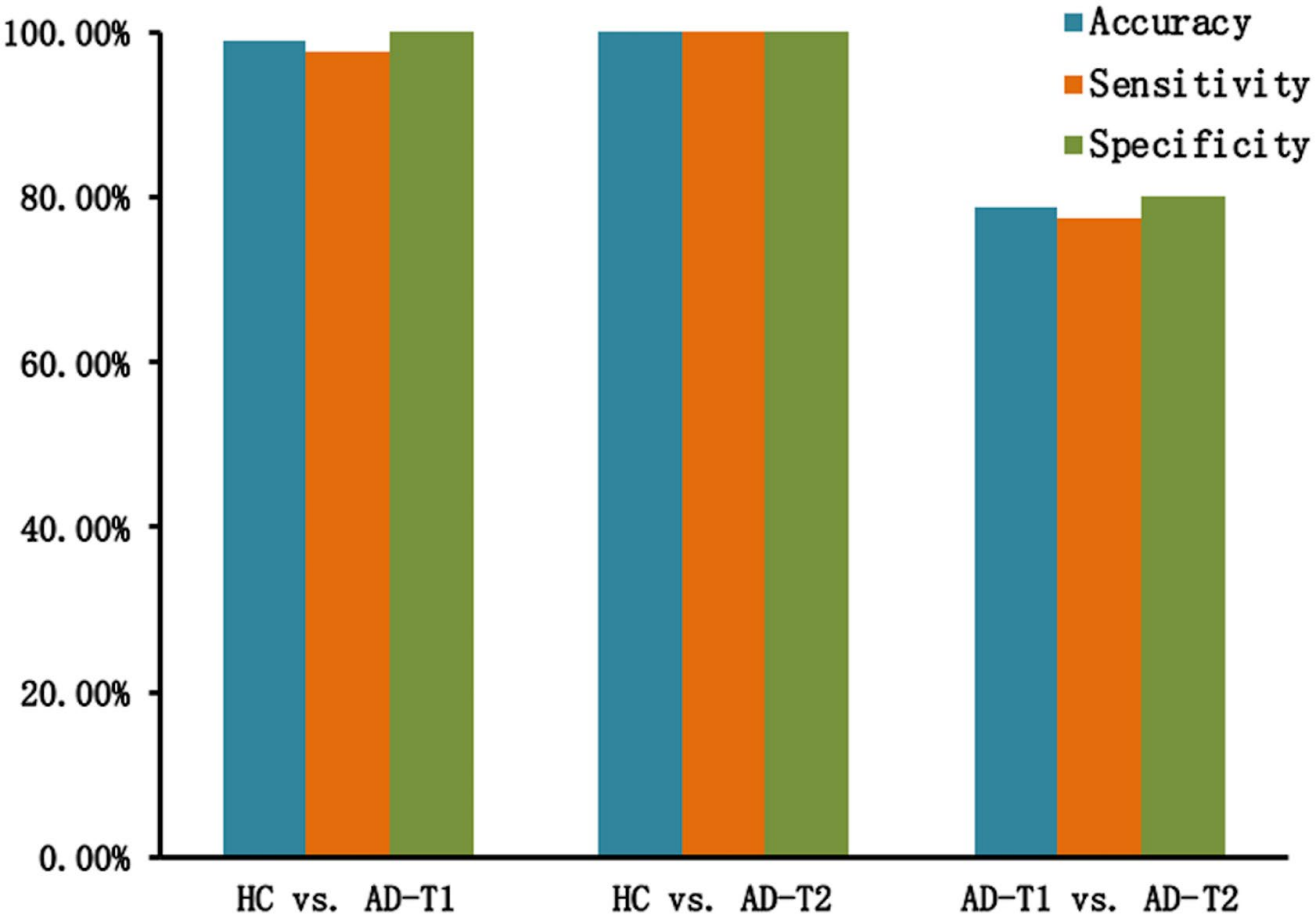
Classification results. There are high classification accuracy, sensitivity, and specificity for HC vs. AD-T1 (accuracy of 98.82%, sensitivity of 97.5%, and specificity of 100%), HC vs. AD-T2 (accuracy, sensitivity, and specificity of 100%), and AD-T1 vs. AD-T2 (accuracy of 78.75%, sensitivity of 77.5%, and specificity of 80%).

For the classification analysis, 5 features including mean GMV values within overlapped decrease areas, continuing decrease areas, left and right STG, and left caudate were entered the prediction model. As displayed in Figure 7, the SVM classification analysis applying the LOOCV method achieved: (1) accuracy of 98.82%, sensitivity of 97.5%, and specificity of 100% for HC vs. AD-T1; (2) accuracy, sensitivity, and specificity of 100% for HC vs. AD-T2; (3) accuracy of 78.75%, sensitivity of 77.5%, and specificity of 80% for AD-T1 vs. AD-T2.

## Discussion

In the present study, we investigated grey matter atrophy in AD patients over a one-year period using a grey matter based spatial statistics approach (Parvathaneni et al., 2017), i.e., measures of grey matter volume on the cortical skeleton (Ball et al., 2013; Ouyang et al., 2019). We observed reduced total GMV and grey matter atrophy in a wide range of regions including inferior frontal gyrus, prefrontal cortex, lateral temporal gyrus, posterior cingulate cortex, insula, hippocampus, caudate, and thalamus in AD patients at both time points as compared to HC. We also found decreased GMV in bilateral STG and left caudate between AD-T2 and AD-T1, which was significantly correlated with concurrent cognitive decline (i.e., change scores in the MoCA). Further, we reported reduced structural covariance connectivity between right STG and left caudate in AD patients vs. HC, and significant causal effects of overlapped decrease areas in AD-T1 on continuing decrease areas, left and right STG, and left caudate in AD-T2. The classification analysis with AD-related grey matter atrophy as features demonstrated high accuracy, specificity, and sensitivity in classifying AD patients from HC, and AD-T1 from AD-T2. These findings provide new evidence for progressive brain structural abnormalities in AD patients over a one-year period, and associations between brain atrophy and cognitive decline in AD progression.

We observed that AD patients showed substantial grey matter atrophy in a wide range of brain regions, including inferior frontal gyrus, prefrontal cortex, lateral temporal gyrus, posterior cingulate cortex, insula, hippocampus, caudate, and thalamus. Most of these regions are consistent with those reported in previous studies (Karas et al., 2003; Zakzanis et al., 2003; Yang et al., 2012; Wang et al., 2015). However, in the current study, using the grey matter based spatial statistics, we also observed significant GMV differences in inferior frontal gyrus and insula. It may suggest that measurement of GMV at the core of the cortical plate is more sensitive to detecting group differences between AD patients and HC (Parvathaneni et al., 2017) as it alleviates partial volume effect and reduces individual variability (Ouyang et al., 2019).

One of the key findings is that we found significant decrease in the volume of bilateral STG and left caudate in AD patients over a one-year period. This finding of significant decrease in the volume of bilateral STG is consistent with that reported in a previous longitudinal study in AD patients showing fast atrophy in temporal lobes with time (Dicks et al., 2019). Reduced volume of template lobe and caudate has been found in a subtype of AD (i.e., Dominantly-Inherited Alzheimer’s Disease) as compared to noncarriers (Cash et al., 2013; Ryan et al., 2013). The significant reduction in the volume of caudate has also been observed in both MCI and AD patients as compared to normal adults (Barber et al., 2002; Almeida et al., 2003; Madsen et al., 2010; Jiji et al., 2013), while there was one study that reported no obvious change in the volume of caudate until the moderate stage of AD (Roh et al., 2011). Further, in agreement with previous studies (Looi et al., 2008; Liu et al., 2010; Jiji et al., 2013), we found significant correlations between change values of GMV in left caudate and cognitive decline in AD patients. For example, higher GMV values within caudate were correlated with higher cognitive function as indexed by MMSE scores across AD patients and control adults (Looi et al., 2008). Changes in the volume of caudate served as an indication of early AD (Jiji et al., 2013) and as predictors of the conversion from MCI to AD (Liu et al., 2010). Put together, these findings suggest STG and left caudate might be important neuroanatomical markers of pathological progression of AD.

In addition to AD-related GMV decrease, we also observed significantly reduced structural covariance connectivity between right STG and left caudate in AD patients as compared to HC. The structural covariance connectivity has been suggested as an effective method to comprehensively investigate different networks related to AD (Montembeault et al., 2016; Li et al., 2019). A few studies have demonstrated abnormal structural covariance networks in AD patients as compared to HC (Hafkemeijer et al., 2016; Montembeault et al., 2016; Chang et al., 2018, 2021; Li et al., 2019). For example, a recent study explored the grey matter SCN along AD continuum and reported reduced SCN between DMN and salience network associated with AD neuropathological progression (Li et al., 2019). Here, we present significantly reduced structural covariance connectivity between right STG and left caudate in AD patients as compared to HC, but no differences between AD-T2 and AD-T1. However, there were significant causal effects of overlapped decrease areas in AD patients at baseline (AD-T1) on continuing decrease areas, bilateral STG, and left caudate in AD patients at one-year follow-up (AD-T2). These findings suggest grey matter structural covariance connectivity is valuable in providing supplementary information linked to progressive changes in AD patients. However, given the relatively small sample size in the current study, further research is needed to confirm these results.

Another interesting findings are the classification results based on AD-related grey matter atrophy. Applying a linear SVM approach, it reached high accuracy, specificity, and sensitivity in classifying HC and AD patients, and AD patients at different time points. Previous studies have suggested that the pattern of GMV decrease is potentially useful for the classification of AD from cognitive normal adults (CN) (Magnin et al., 2008; Guo et al., 2014; Kruthika et al., 2018). Indeed, including overlapped and continuing decrease areas and regions showing progressive decrease in AD patients as features of the classification analysis, there were high discrimination accuracy, sensitivity, and specificity in classifying AD and CN, and classifying AD-T1 and AD-T2, suggesting patterns of GMV decrease serve as sensitive features of identifying the pathological progression of AD.

There are two limitations worth noting when interpreting the results. First, to obtain a reasonable amount of longitudinal MRI data, we included MRI scans collected in multi-sites and different models (i.e., Verio, TrioTim, and Skyra) of SIEMENS 3.0 T scanners. And, to minimize the inhomogeneity of the imaging data, we only included data scanned with the same parameters, which limited the sample size in the present study. In future studies, MRI data with larger sample sizes are needed to confirm the findings reported here. Second, structural covariance connectivity did not allow for the assessment of connectivity strength at the individual level, so it is still unclear how the abnormal structural covariance connectivity relates to the cognitive decline in AD patients. Thus, it would be worth examining the relationship of structural covariance connectivity and cognitive function to further characterize the neuroanatomical changes underlying pathological progression in AD.

## Conclusion

In conclusion, using a grey matter based spatial statistics, we observed that AD patients at both time points showed grey matter atrophy in inferior frontal gyrus, prefrontal cortex, lateral temporal gyrus, posterior cingulate cortex, insula, hippocampus, caudate, and thalamus as compared to HC. There was also significant GMV decrease in both bilateral STG and left caudate in AD patients over a one-year period, and GMV decrease in right STG and left caudate was positively correlated with the concurrent cognitive decline. Further, AD patients demonstrated reduced structural covariance connectivity between right STG and left caudate, and overlapped decrease areas in AD-T1 had causal effects on continuing decrease areas, bilateral STG, and left caudate in AD-T2. In addition, using patterns of AD-related GMV decrease as features, there were high discrimination accuracy, specificity, and sensitivity in classifying HC and AD patients, and classifying AD patients at different time points. Our study provides novel insights into the structural deterioration related to pathological progression of AD and highlights the role of STG and left caudate in cognitive decline in AD patients. These findings may serve as a clinical aid to the prognosis of AD progression.

## Data availability statement

Data used in the present study were obtained from the Alzheimer’s Disease Neuroimaging Initiative (ADNI) database (adni.loni.usc.edu). The ADNI was launched in 2003 as a public-private partnership led by Michael W. Weiner. The primary goal of the ADNI is to test whether magnetic resonance imaging (MRI), positron emission tomography (PET), other biological markers, and clinical and neuropsychological assessment can be combined to measure the progression of mild cognitive impairment (MCI) and early Alzheimer’s disease (AD). For more information, please visit www.adni-info.org. The tidy data used in the current study are available at https://github.com/Yaqiongxiao/Progressive.changes.GM.AD.

## Author contributions

YX and JW contributed to the conception and design of this study. KH and YX contributed to the data acquisition from ADNI. JW contributed to the data analysis. YX drafted the manuscript. JW, LG, and SY reviewed the manuscript. All authors contributed to the article and approved the submitted version.

## Funding

This work was supported by the Shenzhen Science and Technology Innovation Commission (JCYJ20200109144801736), the National Natural Science Foundation of China (32200808, 82001799), and the Natural Science Foundation of Yunnan Province (202001BC070001, 202102AA100053).

## Conflict of interest

The authors declare that the research was conducted in the absence of any commercial or financial relationships that could be construed as a potential conflict of interest.

## Publisher’s note

All claims expressed in this article are solely those of the authors and do not necessarily represent those of their affiliated organizations, or those of the publisher, the editors and the reviewers. Any product that may be evaluated in this article, or claim that may be made by its manufacturer, is not guaranteed or endorsed by the publisher.

## Code availability

R and matlab code for implementing all the analyses can be found at https://github.com/Yaqiongxiao/Progressive.changes.GM.AD.

## Footnotes

^1^ https://adni.loni.usc.edu/

^2^ https://adni.loni.usc.edu

^3^ http://fsl.fmrib.ox.ac.uk/fsl/fslwiki/TBSS
